# A Multi-Objective Multi-Label Feature Selection Algorithm Based on Shapley Value

**DOI:** 10.3390/e23081094

**Published:** 2021-08-22

**Authors:** Hongbin Dong, Jing Sun, Xiaohang Sun

**Affiliations:** Department of Computer Science and Technology, Harbin Engineering University, Harbin 150001, China; donghongbin@hrbeu.edu.cn (H.D.); sunxiaohang@hrbeu.edu.cn (X.S.)

**Keywords:** multi-label learning, feature selection, multi-objective optimization, Shapley value

## Abstract

Multi-label learning is dedicated to learning functions so that each sample is labeled with a true label set. With the increase of data knowledge, the feature dimensionality is increasing. However, high-dimensional information may contain noisy data, making the process of multi-label learning difficult. Feature selection is a technical approach that can effectively reduce the data dimension. In the study of feature selection, the multi-objective optimization algorithm has shown an excellent global optimization performance. The Pareto relationship can handle contradictory objectives in the multi-objective problem well. Therefore, a Shapley value-fused feature selection algorithm for multi-label learning (SHAPFS-ML) is proposed. The method takes multi-label criteria as the optimization objectives and the proposed crossover and mutation operators based on Shapley value are conducive to identifying relevant, redundant and irrelevant features. The comparison of experimental results on real-world datasets reveals that SHAPFS-ML is an effective feature selection method for multi-label classification, which can reduce the classification algorithm’s computational complexity and improve the classification accuracy.

## 1. Introduction

Classification is an important technical task in pattern recognition. Traditional supervised learning mainly involves single-label classification. However, real-world problems are more complicated. Every sample is labeled with multiple labels. In order to study such objects, research on multi-label learning has emerged over the years. Multi-label learning methods were first used in text classification [1], and they have been applied to new applications such as image annotation [2,3], and biological information [4,5] with the development of research.

The process of multi-label learning is difficult, the size of the label set is uncertain and there is a certain correlation between the labels [6]. Multi-label learning methods are roughly divided into two categories: problem conversion and algorithm adaptation [7]. The original data is converted to problems that can be solved with single-label classifiers in the problem conversion method, such as label power-set method (LP) and binary relevance method (BR) [8]. The algorithm adaptation method does not need to transform the original data but improves the single-label learning methods to adapt to multi-label data, such as a lazy learning approach (MLKNN) [9] and a kernel method (RankSVM) [10]. The algorithm adaptation method does not destroy the original data and better consider the correlation between labels.

The combination of features is critical to the quality of the classification results. The original feature set may contain redundant features and irrelevant features. If the original features are directly input into the classifier, it may interfere with the classification decision of the classifier [11]. The objective of feature selection is to remove redundant features and irrelevant features, thereby reducing the dimensionality of the data and improving the classification accuracy [12]. Therefore, feature selection is able to avoid the disaster of dimensionality in single-label and multi-label data.

At present, most of the feature selection research focuses on single-label learning and has been integrated into practical problems. For example, Stai et al. used a weighted network graph structure to represent multimedia content features and feature weights [13]. The proposed framework structure can identify effective features through a relevance feedback mechanism and provide suitable recommendations. Hs et al. proposed a novel feature selection method that combines three different measurements. This method can qualitative information and can be effectively applied to intrusion detection scenarios [14]. Rauber et al. employed a feature extraction algorithm to extract features with strong discriminative information for bearing fault classification, and then used a greedy algorithm to remove irrelevant and redundant features. This framework has the potential to be extended to the industrial field [15].

Similarly, feature selection methods for multi-label data roughly fall into filter model, wrapper model and embedded model [16]. The filter model does not include a classifier in the evaluation process [17]. Different measurement methods are used to mine feature and label information, such as information entropy [18], correlation [19] and consistency measures [20]. Researchers apply the idea of feature selection study for single-label learning to multi-label task and many excellent algorithms such as multi-label feature selection based on max-dependency and min-redundancy (MDMR) [21], information gain based on the BR approach (IG_BR) [22] and multi-label informed feature selection method (MIFS) [6] are proposed. They are extensions of feature selection based on the maximum relevance and minimum redundancy criterion (mrmr) [23], feature selection via maximizing global information gain (IG) [24] and feature selection based on mutual information (MI) [25] respectively. There are also feature selection methods specifically designed for multi-label learning, such as manifold-based constraint Laplacian score (MCLS) [26] and manifold regularized discriminative multi-label feature selection algorithm MDFS [27]. Embedded model completes the feature reduction in multi-label learning [28,29].

Compared with filter feature selection, the wrapper type is better in classification accuracy, because the classification result is directly utilized to assess the feature subsets [30]. The evolutionary-based feature selection algorithms are popular because evolutionary algorithms have strong global optimization capabilities and can consider the combination of features. In feature selection, smaller-scale features are expected to obtain higher classification accuracy. However, traditional evolutionary algorithms can only set one fitness function, so multi-objective evolutionary algorithms have attracted attention, which can balance the relationship between multiple objectives [31]. The relationship between these objectives may be contradictory. Different from single-label learning, the assessment indicators of multi-label learning are intricate. Therefore, the classification accuracy criterion is specially used for multi-label data in the wrapper multi-label feature selection optimization algorithms.

A wrapper feature selection algorithm is proposed to decrease the features of the original multi-label samples in this paper. A classic multi-objective algorithm which is called the third generation of non-dominated genetic algorithm (NSGA III) [32] is employed to optimize three objectives of Hamming loss, average precision, and the scale of feature subset. The original NSGA III was first proposed for consecutive applications, but feature selection corresponds to the discrete optimization scenario. To cope with this problem, we have made improvements on the encoding method, crossover operator and mutation operator of NSGA III. In order to effectively identify irrelevant and redundant features, we introduce Shapley value to assess the contribution of the features and propose a crossover operator and a mutation operator based on Shapley value to make the global search and local search in a balanced state, thereby increasing the accuracy of the classifier results and improving the algorithm’s convergence speed. The main contributions of this work are presented as follows:

- Shapley value and multi-objective multi-label feature selection are fused from two sides: feature and individual.
- Two improved operators are proposed, which adaptively adjust the crossover and mutation probability by evaluating the features’ contribution and equate the algorithm’s global and local search.
- An improved archive maintenance strategy is put forward to increase the convergence performance of the multi-objective optimization method.
- Experiments on datasets of different scales prove the validity and adaptability of the proposed algorithm.

The rest of the paper is presented as follows: Section 2 analyzes the research status of multi-label feature selection and Shapely value in feature selection. Section 3 describes the basic knowledge of multi-objective optimization, Shapley value and multi-label learning. Section 4 introduces the objective functions, mutation operator, crossover operator, archive set maintenance strategy and the flow chart of the proposed algorithm SHAPFS-ML. Section 5 shows the experimental results on seven multi-label datasets. Section 6 gives a summary of this paper.

## 2. Related Works

The search ability of feature selection algorithm is an important factor that determines the quality of selected feature subsets. The exhaustive search selects the optimal feature subset by enumerating the possible combinations of features. Mnich et al. used multidimensional exhaustive analysis of the mutual information between features and labels [33]. This method requires a large number of samples and has a high computational cost. Although exhaustive searching can find the global optimal solution, it is inefficient. Heuristic search uses heuristic information to reduce the search range of the feature space. Hua et al. proposed an improved modified strong approximate Markov blanket method to remove redundant features, and then used sequential forward selection (SFS) method to remove irrelevant features [34]. Fa et al. proposed a backward selection (SBS) approach to eliminate a set of features that are not helpful for classification [35]. Both SFS and SBS are greedy algorithms which selects the current best solution and are can easily fall into the local optimum. Evolutionary computing technology belongs to the heuristic search. Such methods use a random search with heuristic information, which can obtain approximate optimal solutions. Common evolutionary computing methods include genetic algorithm (GA), particle swarm optimization (PSO), differential evolution (DE), etc. Therefore, we use evolutionary computing theory as the methodology in this paper.

In recent years, multi-objective optimization has been a research hotspot in the field of evolutionary computing, and it has been successfully applied to solve the problem of feature selection. Bing et al. proposed a multi-objective differential evolution algorithm to optimize the two objectives of reducing the number of selected features and the classification error rate [36]. Experimental results showed that the proposed algorithm can give a more trade-off solution set and improve the quality of the solution compared with single-objective optimization methods. Liam et al. proposed a binary multi-objective PSO algorithm for filter feature selection based on rough set theory [37]. The performance of this method is better than the traditional single-objective PSO. Nouri-Moghaddam et al. used a novel forest optimization algorithm (FOA) algorithm and designed multiple concepts to deal with the feature selection problem in a multi-objective optimization manner [38]. The experimental results showed that the proposed method performs better than other single-objective and multi-objective optimization methods.

Nowadays, compared with multi-label feature selection algorithms, there are more studies on single-label feature selection algorithms [39]. The investigation on multi-objective optimized multi-label feature selection algorithm has been proposed only recently, benefitting from the successful application of multi-objective optimization methods in single-label feature selection. In 2014, Yin et al. analyzed the contradictions between the two types of multi-label classification indicators and used the second generation of non-dominated genetic algorithm (NSGA II) to optimize Hamming loss and average precision [40]. The proposed algorithm performed better compared with other methods. In 2017, Zhang et al. presented a multi-objective particle swarm optimization method to cover the multi-label feature selection problem [41]. In order to enhance the multi-objective optimization algorithm’s performance, they proposed two operation operators. And the crowding distance mechanism was used for the maintenance of archives. The results showed the exploration ability of the proposed algorithm is better than that of NSGA II. In 2021, Bidgoli et al. proposed a discrete differential evolution method for multi-label feature selection and proposed a binary mutation operator to improve the multi-objective optimization’s global search capabilities [42]. The proposed method’s performance is verified from the assessment of the multi-objective algorithm and the accuracy for classification.

To assess features’ importance for classification, Shapley value in cooperative game theory is introduced. In 2005, Cohen et al. put forward a feature selection algorithm using the Shapley value. It utilized the Shapley value to iteratively calculate the validity of the feature, and features were selected through forward and backward elimination [43]. The forward elimination method achieved the highest accuracy among the experimental comparison algorithms. In 2016, Mokdad et al. designed a feature selection algorithm structure derived from the Shapley value [44]. First, the rank of N groups of features was obtained by N feature selection algorithms, and then the Borda Coun method was adopted to determine the ultimate feature rank. The experimental results showed the validity of this algorithm. In 2020, Chu et al. decomposed the Shapley value into high-order interactive components to reasonably evaluate features’ contribution and proposed to evaluate feature subsets by discarding unselected features [45]. The above methods are non-optimized algorithms. Some studies combine the Shapley value with evolutionary algorithms. Deng et al. put forward a feature selection method that combines the Shapley value and particle swarm optimization [46]. The Shapley value was utilized to remove useless features in the local search and select fewer features. Guha et al. proposed a cooperative genetic algorithm for feature selection [47]. The fitness function combined the classification result, the feature subset size and the Shapley value score in a multi-objective fashion. However, this method is essentially single-objective optimization and cannot obtain multiple non-dominated solutions.

As far as we know, Shapley value has not been introduced into the multi-label feature selection algorithm. We merge Shapley value and a multi-objective multi-label feature selection method. This combination possesses the following two advantages: First, the Shapley value method focuses on the contribution of each feature, and the multi-objective optimization method focuses on the combination of features. Appropriate fusion can prevent a feature from being eliminated due to its poor performance in the feature subset, but the feature is useful for classification. Second, due to the huge search space, the search process at the beginning of the evolutionary algorithm has some randomness. The Shapley value method is conducive to the algorithm to search for potential spaces and to improve the convergence speed.

## 3. Preliminaries

### 3.1. Multi-Objective Optimization

In reality, many optimization problems involve multiple objectives, moreover, there may be contradictions or other relationships between the objectives. It is hard for people to acquire the best solution for each objective and determine the importance of different objectives. The multi-objective algorithm can balance the relationship between multiple objectives so that the obtained solution can be approximately optimal on multiple objectives.

The multi-objective optimization problem with M optimization objectives is formally defined as Equation (1):

(1) $$minimizef\;\left(x\right)\;=\;\left(f_{1}\left(x\right),f_{2}\left(x\right),\ldots f_{M}\left(x\right)\right)$$

where x∈Ω, Ω represents the decision space, $x\;=\;\left(x_{1},\;x_{2},\ldots ,x_{n_{m}}\right)\;\in \;\Omega \;\subseteq \;{\mathbb{R}}^{n}$, x is a decision variable of length $n_{m}$, $f_{i}\left(x\right)\;\left(i=1,\ldots ,M\right)$ is the *i*-th optimized function.

In a multi-objective optimization problem, for two solutions y,z∈Ω, *y* dominates *z*, donated by *y* ≺ z, if ∀k: $f_{i}\left(y\right)\;\leq \;f_{i}\left(z\right)\;\wedge \;\exists k:\;f_{i}\left(y\right)\;<\;f_{i}\left(z\right),\;k\;\in \;\left[1,M\right]$. $x^{*}\in \Omega$ is defined as a Pareto optimal solution if there is no other solution $x\;\neq \;x^{*}\;\in \;\Omega$ dominates $x^{*}$. A Pareto set consists of all the Pareto optimal solutions. The front obtained by mapping the Pareto set to the objective space is known as the Pareto front.

### 3.2. Shapley Value

Game theory mainly includes two types of cooperative games and non-cooperative games [48,49]. The main feature of cooperative games is that participants cooperate with each other and form alliances to maximize the overall benefits. The collective benefits are more important than the individual benefits. Cooperative games emphasize collective rationality [50], while non-cooperative games emphasize individual rationality [51]. Feature selection can be regarded as a cooperative game, which satisfies the forming conditions of the cooperative game:

(1) The total personal income is less than the alliance’s income.
(2) Compared with not joining the alliance, every participant is able to gain a higher profit.

The Shapley value calculates the weighted sum of the participants’ marginal contributions in the cooperative game [52]. It’s a fair and reasonable method of distributing benefits for participants. In feature selection, Shapley value can be utilized to calculate the feature’s contribution.

Suppose that the set of individuals participating in the cooperative game is $P\;=\;\left\{p_{1},\;p_{2},\ldots ,p_{n_{s}}\right\}$, $p_{i}$ is the *i*-th participant, and $n_{s}$ is the number of individuals. S is the set of all subsets that do not contain $p_{i}$ in P. v is a real-valued function, which can map the alliance to the benefits obtained by the cooperation of participants in the alliance. The Shapley value of participant $p_{i}$ is calculated as follows:

(2) $$\phi_{i}=\sum_{S\subseteq P\backslash \left\{p_{i}\right\}}\frac{\left|S\right|!\left(\left|P\right|-\left|S\right|-1\right)!}{\left|P\right|!}\left[v\left(S\cup \left\{p_{i}\right\}\right)-v\left(S\right)\right]$$

Specifically, in the feature selection problem, P is the original feature set, $p_{i}$ is the *i*-th feature, *S* is all feature subsets that do not contain feature $p_{i}$, and the function *v* is represented by the classification result of the selected features under the classifier.

### 3.3. Multi-Label Learning

**Figure 1:**
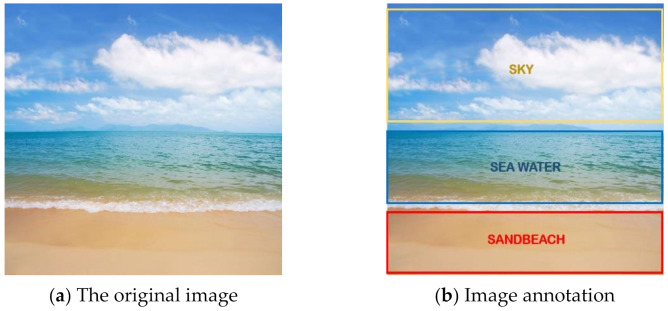
An example of multi-label classification.

In order to better illustrate the difference between multi-label learning and traditional single-label learning, we give an example of an image. As shown in Figure 1, (a) is the original image, and (b) is the annotated image of (a). First, the meaning of the traditional single-label classification is explained. When we judge that the water area in (a) is a sea, a lake or a river, we can see that it belongs to the sea, and it cannot belong to the lake and the river at the same time, because these three categories are in conflict. This problem is a single-label multi-classification problem, that is, there are three categories of sea, lake and river under the label of water description. However, an image often contains more than one object. As shown in (b), the image can be annotated with three labels including sky, sea and sand beach. Each label can be divided into two categories, 0 and 1, meaning including the object and not including the object. Obviously, these two categories are contradictory. However, the three labels can exist at the same time, and there is a certain connection between the three labels. For example, if an image contains sea water, then the image may also include sky and sand beach, which is in line with the objective laws of the world. Therefore, multi-label classification is close to real life. The classification in (b) can be regarded as a multi-label binary classification problem. The multi-label multi-class problem corresponds to multiple labels, and each label has multiple categories of problems.

The original single-label learning method cannot be directly used in multi-label learning [53], because every sample of multi-label data is labeled with one or more labels simultaneously. Moreover, the relationship between the labels may be related. The definition of multi-label learning is as follows:

Let $X=\left\{x_{1},x_{2},\ldots ,x_{N}\right\}$ be the d-dimensional input variable on the real number field, and $Y=\left\{y_{1},y_{2},\ldots ,y_{q}\right\}$ be the label space. $D=\left\{\left(x_{i},Y_{i}\right),1\leq i\leq N\right\}$ is the training dataset, and $Y_{i}$ is the true label set of training data $x_{i}$. During the training process, the algorithm learns the function $h:X\to \;2^{q}$ based on the training data. Given test data set $H=\{\left(x_{j},y_{j}\right)|1\leq j\leq t\}$, when the test data $x_{j}\in X$ is input, the predicted labels closest to the proper labels of $x_{j}$ are obtained through the function h.

Multi-label classification has unique evaluation means to analyze the quality of the classification results, which is divided into examples-based criteria and labels-based criteria [54]. In this paper, we mainly introduce the six criteria involved in the experiment:

- Ranking Loss: It evaluates the fraction that an irrelevant label is ranked before a related label. $\bar{y_{i}}$ is the complementary set of $y_{j}$.
  (3) $$\begin{array}{c} RL\left(h,H\right)=\frac{1}{t}\overset{t}{\underset{j=1}{\sum }}\{\frac{1}{\left|y_{j}\right|\left|\bar{y_{i}}\right|}|\left(k,l\right)\in \left(y_{j}\times \bar{y_{i}}\right),s.t.h\left(x_{j},k\right)\;\leq \;h\left(x_{j},l\right)\} \end{array}$$
- Average Precision: It measures the average instances’ correlated labels, and these labels are ranked higher than the preset labels. $rank_{h}$ is the descending rank function.
  (4) $$\begin{array}{c} \;AP\left(h,H\right)=\frac{1}{t}\overset{t}{\underset{j=1}{\sum }}\frac{1}{\left|y_{j}\right|}\underset{y\in y_{j}}{\sum }\frac{\left|\left\{y^{\prime }|rank_{h}\left(x,y^{\prime }\right)\leq \;rank_{h}\left(x_{j},y\right),y^{\prime }\in y_{j}\right\}\right|}{rank_{h}\left(x_{j},y\right)} \end{array}$$
- Coverage: It records the minimum number of steps that need to be moved to cover the true labels associated with the sample from the sample’s classification prediction labels list.
  (5) $$\begin{array}{c} CV\left(h,H\right)=\frac{1}{t}\overset{t}{\underset{j=1}{\sum }}max_{y\in y_{j}}rank_{h}\left(x_{j},y\right)-1 \end{array}$$
- Hamming loss: It measures the proportion of misclassified label pair.
  (6) $$\begin{array}{c} HL\left(h,H\right)=\frac{1}{t}\overset{t}{\underset{j=1}{\sum }}\left|h\left(x_{j}\right)⨁y_{j}\right| \end{array}$$
  where ⨁ represents the symmetric difference of the predicted label set and true label set.
- Macro-F1: It is a label-based index that takes into account the average F-measure of every label.
  (7) $$\begin{array}{c} MaF\left(f,F\right)=\frac{1}{q}\overset{q}{\underset{j=1}{\sum }}\frac{2\sum_{i=1}^{t}y_{ij}h_{j}\left(x_{i}\right)}{\sum_{i=1}^{t}y_{ij}+\sum_{i=1}^{t}h_{j}\left(x_{i}\right)} \end{array}$$
- Micro-F1: It is a label-based index that takes into account the average F-measure of the prediction matrix.
  (8) $$\begin{array}{c} MiF\left(h,H\right)=\frac{2\sum_{j=1}^{t}\left|h\left(x_{j}\right)\cap y_{j}\right|}{\sum_{j=1}^{t}\left|y_{j}\right|+\sum_{j=1}^{t}\left|h\left(x_{j}\right)\right|} \end{array}$$

## 4. The Proposed Method

### 4.1. Objective Function

Generally, the number of objectives for multi-objective optimization does not exceed three, and problems with more than three objectives can be defined as many-objective optimization problems. This paper sets three optimization objectives including AP, HL and the number of selected features. The calculation methods of AP and HL are shown in Equations (4) and (6). The larger the value of AP, the better, and the smaller the value of HL and the number selected features, the better. The relationship between three objectives is complicated. First, the classification index and the number of features are contradictory in most situations. It is difficult to obtain high classification accuracy with a small number of features. Second, the literature [40] pointed out that AP and HL are contradictory. The single-objective optimization method is difficult to deal with the complex relationship between the objectives, so the multi-objective optimization algorithm is adopted as the basic optimization method.

### 4.2. Mutation Operator

Like the traditional genetic algorithm, NSGA III requires the crossover and mutation operators to produce offspring. Traditional NSGA III suites for continuous optimization matters, which means that the algorithm uses the real number encoding method and operators such as simulate binary crossover and polynomial mutation. Feature selection is a discrete optimization problem, and every feature corresponds to a bit. In the genetic algorithm, for a dataset with d-dimensional features, the population is composed of multiple chromosomes composed of 0 and 1 with length d, and each chromosome represents a solution. 0 means the feature corresponding to the bit is not selected, 1 means selected.

In order to effectively identify the relevant features, redundant features and irrelevant features, the Shapley value is fused with the multi-objective optimization algorithm. A mutation operator and a crossover operator based on Shapley value are proposed so that the improved NSGA III algorithm can resolve the multi-label feature selection problem well.

In the real world, many problems involve with high dimensions. The high-dimensional characteristics of the data increase the decision variables of the evolutionary algorithm, resulting in a huge search space, and the algorithm’s optimization ability and speed are affected. At the beginning, the algorithm’s randomness may lead to the wrong search direction for certain features. For example, suppose that there are m individuals in a population, the length of each individual is d, the *i*-th feature is relevant, and the *j*-th feature is irrelevant. Then in the early stage of the iteration, the following two situations may occur:

(a) The number of individuals who choose the *i*-th feature is u, $\frac{m}{2}\;\leq \;u\;<\;m$, and the number of individuals who do not choose the *i*-th feature is m−u.
(b) The number of individuals who choose the *j*-th feature is w, $\frac{m}{2}\;\leq \;w\;<\;m$, and the number of individuals who do not choose the *j*-th feature is m−w.

When u and w are large, it indicates that most individuals have not selected the relevant feature i, or that most individuals have selected the irrelevant feature j. The offspring of similar chromosomes are close to the parent individuals. In this case, it is requisite to carry out mutation operations on the bits with the above-mentioned conditions with greater probability.

However, it is difficult to know the relevance or irrelevance of features. Therefore, we introduced the Shapley value to evaluate the feature’s contribution. Given that three objectives are optimized in this paper, including two multi-label evaluation criteria, the Shapley value of the feature is defined as follows:

(9) $$\begin{array}{c} \phi_{i}=\sum_{S\subseteq P\backslash \left\{p_{i}\right\}}\frac{\left|S\right|!\left(\left|P\right|-\left|S\right|-1\right)!}{\left|P\right|!}\left[v_{1}\left(S\cup \left\{p_{i}\right\}\right)-v_{1}\left(S\right)+v_{2}\left(S\cup \left\{p_{i}\right\}\right)-v_{2}\left(S\right)\right] \end{array}$$

where $v_{1}$ is the HL value obtained by the feature subset S under the multi-label classifier, and $v_{2}$ is the AP value. HL and AP are calculated as shown in Equation (6) and Equation (4).

When the unbalanced search situation no longer occurs, it means that the relevant features have basically been selected, and the irrelevant features are discarded. In order to further judge the redundant features, more attention should be paid to the features near the Shapley value of 0. Because the contribution of such features hardly contribute to classification, whether to choose these features basically does not affect the classification result. The specific mutation procedure is shown in Algorithm 1.
**Algorithm 1** Mutation probability calculation**Input:** Population pop; Population scale $N_{p}$; Feature dimension *d*; Default mutation rate η; Parameter maker *t*.**Output:** Mutation probability
$P_{mu}$
1: for i = 1:$\;N_{p}$2:$\;A\left(i\right)\;$= Fit($pop\left(i\right)$);//Calculate the fitness of every individual in pop.3: end4: for i = 1: d5:$\;\varphi_{i}\;$= Shapley(A);//Calculate the Shapley value of the i-th feature.6: number1(i) = Select(pop(:,i));//Record the number of individuals with selected the i-th feature. 7: number2(i) = Unselect(pop(:,i));//Record the number of individuals with unselected the i-th feature.8: end9: for *i* = 1: *d*10: if *t* = 011: if $\varphi_{i}<0$ && (number1(i)/number2(i)) < 1/212:
$P_{mu}\left(i\right)=\eta +\eta *\left(1-\frac{\mathrm{number}1\left(i\right)}{\mathrm{number}2\left(i\right)}\right);$
13: *t* = 1;14: elseif $\varphi_{i}>0$ && (number2(i)/number1(i)) < 1/2 15: $P_{mu}\left(i\right)=\eta +\eta *\left(1-\frac{\mathrm{number}2\left(i\right)}{\mathrm{number}1\left(i\right)}\right)$; 16: *t* = 1;17: else18: $P_{mu}\left(i\right)=\eta$;19: end20: end21: if *t* = 022: if abs$(\varphi_{i}$) < max(abs(φ))/223: $P_{mu}\left(i\right)=\eta +\eta *\left(1-\frac{abs\left(\varphi_{i}\right)}{\max\left(abs\left(\varphi \right)\right)}\right)$;24: else25: $P_{mu}\left(i\right)=\eta$;26: end27: end28: end

Algorithm 1 shows the process of determining the mutation probability of each gene. When a population is initialized, the fitness value of each individual can be calculated through the objective functions (lines 1–3). Then measure the Shapley value of each feature according to Equation (2) and record the number of individuals that selected and unselected a certain feature in the population (lines 4–8). Next, the calculation of mutation probability is divided into two cases. The first is to search for relevant features and irrelevant features under the condition $\left\{\varphi_{i}<0\&\&\left(\frac{\mathrm{number}1\left(i\right)}{\mathrm{number}2\left(i\right)}\right)\leq \;1/2\}|\{\varphi_{i}\;>\;0\&\&\left(\mathrm{number}2\left(i\right)/\mathrm{number}1\left(i\right)\right)\;\geq \;1/2\right\}$. In this case, the *i*-th feature may be wrongly selected or abandoned by most individuals. Therefore, it is necessary to increase the probability of mutation of the *i*-th feature. The more unbalanced the current feature’s search, the greater the probability of mutation. When the population no longer satisfies the above conditions in the later period of evolution, the search for redundant features is performed. The smaller the absolute Shapley value of the feature means the greater the probability that it is a redundant feature, thereby increasing the probability of mutation.

After obtaining the mutation probability of each gene, mutation operation will be performed in the population. The uniform mutation is adopted in this paper, and the specific procedure is shown in Algorithm 2.
**Algorithm 2** Mutation operation**Input:** Population pop; Population mutation ratio $P_{mr}$; Mutation probability $P_{m}$; Population scale $N_{p}$; Feature dimension d.**Output:** Mutation population $pop_{m}$1: $n=N_{p}*P_{mr}$;2: for i = 1: n3: for j = 1: d4: if rand(1) > (1 − $P_{m}\left(j\right)$)5:$\;pop_{m}$(i,j) = abs($pop\left(i,j\right)$ − 1);6: else7:$\;pop_{m}\left(i,j\right)\;$=$\;pop\left(i,j\right)$;8: end9: end10: end

### 4.3. Crossover Operator

The uniform crossover operator is adopted in this paper. The exact process is shown in Algorithm 3.
**Algorithm 3** Crossover operation**Input:** Population pop; Population crossover ratio $P_{cr}$;
 Crossover probability $P_{c}$; Population scale $N_{p}$; Feature dimension *d*.**Output:** Crossover population $pop_{c}$1:$\;n=N_{p}*P_{cr}$;2: m=0;3: for i=1:n4:$\;i1=\mathrm{rand}\left(1,\;n\right)$;//Generate a random number from 1 to n.5: *i2 =*$\;\mathrm{rand}\left(1,\;n\right)$;6: m=2∗i−1;7: for *j* = 1: d8: if rand(1) > rand(0.5,$\;P_{c}$)9:$\;pop_{c}\left(m,j\right)=pop\left(i2,j\right);$10:$\;pop_{c}\left(m+1,j\right)=pop\left(i1,j\right);$11: else12:$\;pop_{c}\left(m,j\right)=pop\left(i1,j\right);$13:$\;pop_{c}\left(m+1,j\right)=pop\left(i2,j\right);$14: end15: end16: end

In the mutation operation, the Shapley value represents a single feature’s contribution. In the crossover operation, we employ Shapley value to evaluate individuals. The average of the sum of the selected features’ Shapley values is defined as the individual’s Shapley value. Given that the Shapley value vector of all the features is φ, and the chromosome of individual i is $pop\left(i,:\right)$, then the Shapley value $\phi_{i}$ of individual i is $\phi_{i}=\frac{pop\left(i,:\right)\times \varphi^{\prime }}{{\left|pop\left(i,:\right)\right|}_{1}}$. The calculation method of the crossover probability $P_{c}$ is as follows:

(10) $$\begin{array}{c} p_{c}=\left\{\begin{array}{cc} 0.5\;, & if\;t=1\\ \frac{\left|\phi_{i1}-\phi_{i2}\right|}{\max\left(\phi \right)-\min\left(\phi \right)}\;, & if\;t=0 \end{array}\right. \end{array}$$

Similar to the mutation probability, the calculation of the crossover probability is divided into two stages. When t=1, the algorithm is in the early stage of the iteration. At this time, the global search of the algorithm is necessary, so the probability of each individual crossover is equal. When t=0, the algorithm performs a local search for redundant features. If individuals with large fitness gaps are selected for crossover, it may affect the inheritance of excellent genes. The non-dominated individuals are at the same level and cannot be ranked in the multi-objective problem. Therefore, we use the individual’s Shapley value to approximately assess the individual’s quality. When *t* = 0, if the difference between the two individuals’ Shapley values is large, then the possibility of swapping genes is reduced.

### 4.4. The Improved Niche Preservation Mechanism

NSGA III employs a set of pre-set reference points to associate non-dominated solutions, and the niche preservation mechanism is employed to select non-dominated individuals from the critical front into the archive. Assume that there are $\rho_{j}$ individuals associated with the *j*-th reference point. The selection process is as follows:

First, randomly select a reference point $\bar{j}$ with the smallest $\rho_{j}$ from the set of reference points. When $\rho_{j}$ = 0, it means that there is no individual associated with it. Therefore, if there is an individual in the critical front that is associated with $\bar{j}$, then the individual closest to the reference line of $\bar{j}$ is selected to the archive. If no individual is associated with $\bar{j}$, then reconsider other reference points.

$\rho_{\bar{j}}\neq 0$ indicates that there are one or more individuals associated with $\bar{j}$. If the number of individuals associated with $\bar{j}$ is zero in the critical front, the next reference point is reconsidered. If the number of individuals associated with $\bar{j}$ is non-zero in the critical front, an individual is randomly selected to associate with $\bar{j}$.

In this paper, we can obtain the Shapley value of each individual, which was introduced in Section 4.2. We sort the individuals in the critical front in descending order according to the individuals’ Shapley value. When $\rho_{\bar{j}}\;\neq \;0$, the first-ranked individual in the critical front is selected and added to the archive if there are individuals in the critical front that are associated with $\bar{j}$. The individual with the highest Shapley value indicates that the features selected by the individual has a higher average contribution to the classification, which means that the individual may be a promising solution. Sorting instead of random selection helps to improve the convergence of the algorithm.

### 4.5. The Overall Flow of the Algorithm

**Figure 2:**
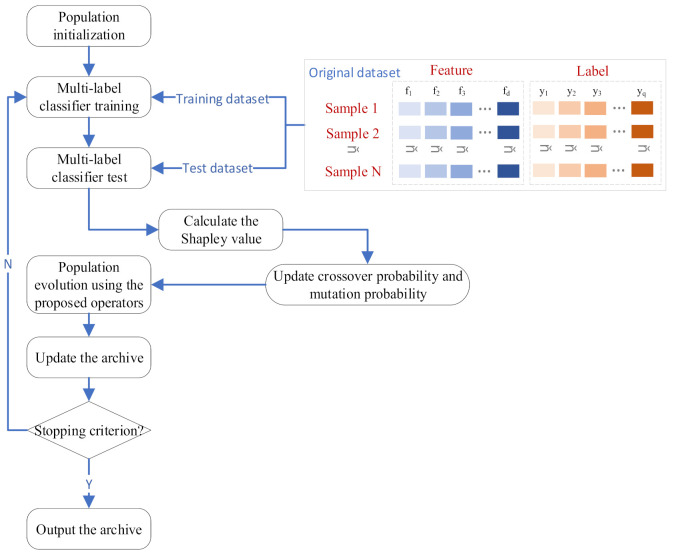
Flow chart of the proposed method SHAPFS-ML.

In order to more clearly illustrate the specific process of the proposed algorithm, Figure 2 shows the flow chart of SHAPFS-ML.

First, the population is initialized. All individuals are binary-coded and the length of the chromosome is the feature dimension of the input data. Then, the fitness values of the individuals in the population are calculated. The multi-label classifier MLKNN is used to evaluate the AP and HL values of the feature subset, and AP, HL and the size of the feature subset are used as the fitness values of the individual. After obtaining the fitness values matrix of the population, the Shapley value of each feature is calculated. Then the crossover probability and mutation probability are determined, and the evolution operation is executed. The regenerated populations are stratified through non-dominated relations. Because the capacity of the archive set is limited, the archive set is maintained by an improved maintenance strategy and the non-dominated solutions in the archive set are updated. If the stop condition is not met, the above process is repeated, and all non-dominated solutions in the archive set are finally output.

We can analyze how the framework is improved from two perspectives. From the perspective of features, each feature acts as a participant to cooperate with other features. The Shapley value can feedback the benefits of the alliances the feature participates in and the alliances that the feature does not participate in. The way of feedback is to adjust the cross probability and mutation probability of the feature, which will affect the probability of the feature appearing in the next iteration, so that the algorithm heuristically searches for potential areas. From the perspective of the population, the fitness function quantifies the quality of each individual. The population evolves through reproduction so that excellent genes are retained in each iteration, disadvantaged individuals are eliminated, and the entire population evolves in a better direction.

## 5. Experiments

### 5.1. Experiment Settings

The experiments are conducted on seven multi-label datasets including flags, emotions, yeast, virus, Languagelog, genbase and medical. Flags, emotions, yeast, genbase and medical are available on MULAN. MULAN is an open-source library for multi-label learning [55]. Languagelog are chosen from MEKA [56], an extended version of WEKA [57] in multi-label learning and evaluation. Virus is available in [58]. Table 1 shows the summary of seven datasets. A classic multi-label classification algorithm ML-KNN [9] is employed as the multi-label classifier. K indicates the number of nearest neighbors, which is set to 10 as suggested in [9]. The parameter η is 0.3 and the size of population is 20. The experiments are conducted on a laptop equipped with an Intel(R) Core (TM) i7-9750H CPU and 16 GB memory.

### 5.2. Comparing Methods

In this section, six comparison methods are employed to demonstrate the usefulness of the proposed algorithm. The traditional NSGA III is compared with SHAPFS-ML to analyze the effectiveness of the improved NSGA III algorithm. Similarly, the coding method of NSGA III is modified to binary, uniform crossover and uniform mutation are adopted as the methods of crossover and mutation. SHAPFS-ML and NSGA III algorithms have been independently run 20 times on each data set. A non-dominated solution set is randomly selected from the running results, and the solution with the smallest sum of all objective function values is selected as the final result. MDFS constructs a low-dimensional embedding method to seek discriminative features [27]. MCLS is a manifold-based method that can transform the original label space and constrain the samples [26]. MIFS exploits the label correlations and decomposes the multi-label information [6]. MDDM maximizes the reliance of features and the associated labels, proj is the irrelated projection dimensionality reduction of MDDM, spc is an unrelated projection feature selection method [59].

### 5.3. Evaluation of Experimental Results on Multi-Label Classification

Table 2, Table 3, Table 4, Table 5, Table 6 and Table 7 show the comparison results on seven datasets under six multi-label learning criteria, which are introduced in Section 3.3. ↑ means the value should be the bigger the better. ↓ means the value should be the smaller the better. Generally speaking, the performance of SHAPFS-ML is the best (In bold). Avg.Rank is the average ranking value of each algorithm on all datasets. The smaller the Avg.Rank value, the better the performance of the algorithm. In detail, SHAPFS-ML has obtained the best results on three indicators average precision, coverage and Hamming loss. According to ranking loss, SHAPFS-ML obtained the optimal results in addition to the dataset Languagelog. SHAPFS-ML ranked second on Languagelog, but the difference between SHAPFS-ML and MCLS is small. On MicroF and MacroF indicators, MCLS is better than SHAPFS-ML on the data set genbase. Although SHAPFS-ML did not rank first on both indicators, SHAPFS-ML is significantly better than other non-optimized methods on other data sets except genbase. For example, on the emotions dataset, the value of SHAPFS-ML on the MicroF indicator is 0.6446, while the values of the other five non-optimized methods are 0.4636, 0.5114, 0.5156, 0.5829 and 0.5860, respectively, which are all behind SHAPFS-ML. Similarly, on the flags dataset, SHAPFS-ML obtained 0.6546 on the MacroF indicator, while in other methods, the lowest value of MDFS is 0.4777, and the highest value of MCLS is 0.5657. This observation reveals that SHAPFS-ML has a remarkable improvement. In terms of average ranking, SHAPFS-ML ranked first, followed by NSGA III. It is observed that the classification results of SHAPFS-ML are improved compared to the traditional NSGA III. Moreover, the multi-objective optimization-based method is more advantageous compared with other non-optimized algorithms, and the performance is relatively stable on different scale datasets. Among the non-optimized methods, MCLS performed best, and MDDM_proj performed worst.

Table 8 shows the ratio of the size of selected features by different methods to the size of the full feature set. It can be seen that SHAPFS-ML can remove at least 60% of the features on the flags, emotions and virus data sets, and can remove more than 50% of the features on other data sets. Although the size of features selected by SHAPFS-ML is not the least, it has the best performance on the classification results. Through the above discussion, we can draw a conclusion that SHAPFS-ML is competitive among the well-established comparison methods.

The average ranking of SHAPFS-ML is better than that of NSGA III under six multi-label learning criteria. The main difference between the two is the crossover and mutation operators. Crossover and mutation operators are related to the global search and local search capabilities of the algorithm. The two types of searches cooperate with each other to achieve a balanced state. There are two main advantages of SHAPFS-ML. First, the crossover and mutation operators proposed in this paper adaptively calculates the crossover probability and mutation probability of the gene locus corresponding to the feature according to the Shapley value during the evolution process. The two operators cooperate and compete with each other to enhance the exploitation of feature space and the ability to explore local features. Secondly, the multi-objective optimization algorithm can consider the combination effect of features, and the introduction of the Shapley value method can measure the effect of a single feature. Feature combinations involving well-performing features may be more competitive and potential. Therefore, in the problem of multi-label feature selection, the optimization ability of SHAPFS-ML is stronger than that of traditional NSGA III. Therefore, the optimization ability of SHAPFS-ML is stronger than that of traditional NSGA III in the problem of multi-label feature selection.

### 5.4. The Comparison on Hypervolume Indicator

To quantify the quality of the Pareto set obtained by SHAPFS-ML, Hypervolume (HV) index is introduced into the evaluation of experimental results. HV is a commonly used index for multi-objective algorithms [60]. The larger the value of HV, the better the multi-objective algorithm’s capability. HV calculates the volume of the hypercube formed by the reference points and the non-dominated solution set. HV value can reflect the distribution and convergence of the algorithm. Therefore, the obtained HV value is different if the non-dominated solution set is different. We have run the algorithms SHAPFS-ML and NSGA III 20 times to calculate the average, best and worst values of HV. As shown in Table 9, SHAPFS-ML can obtain a higher average HV value and the best HV value, which shows that the search ability of SHAPFS-ML’s multi-objective optimization is improved compared with the traditional NSGA III algorithm, and it can obtain a widely distributed and uniform Pareto solution set.

### 5.5. Shapley Value Analysis

To further analyze the validity of the application of Shapley value to multi-label feature selection, we sort the features’ Shapley values calculated in the last iteration of SHAPFS-ML, and gradually select the features for classification according to the order of contribution from the largest to the smallest. NSGA III is a global optimization algorithm, and the number of features cannot be determined arbitrarily. Therefore, NSGA III is not used as a comparison algorithm in this section. The non-optimized algorithms including MDFS, MCLS, MIFS, MDDM_proj and MDDM_spc as mentioned in Section 5.3 are compared for analysis.

The main purpose of this section is to verify whether the Shapley value method can reasonably analyze the contribution of features. This study is meaningful for feature selection. Because features with a high degree of contribution can be regarded as relevant features, which can help the sample to be correctly classified. And features with low contributions can be regarded as irrelevant features, which will interfere with the classification process and may even reduce the classification accuracy. The contribution degree of the feature also reflects the importance of the feature. Other non-optimized methods essentially use different measurement methods to quantify the importance of the feature. Therefore, it is reasonable to compare the feature results based on the Shapley value ranking with other non-optimized methods.

**Figure 3:**
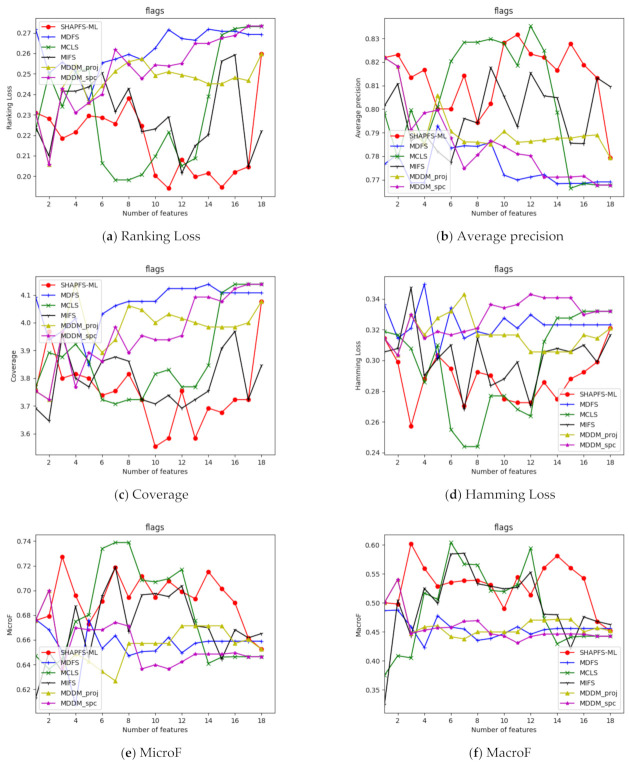
Comparison results with the increase of the number of features under the six indicators on flags dataset.

**Figure 4:**
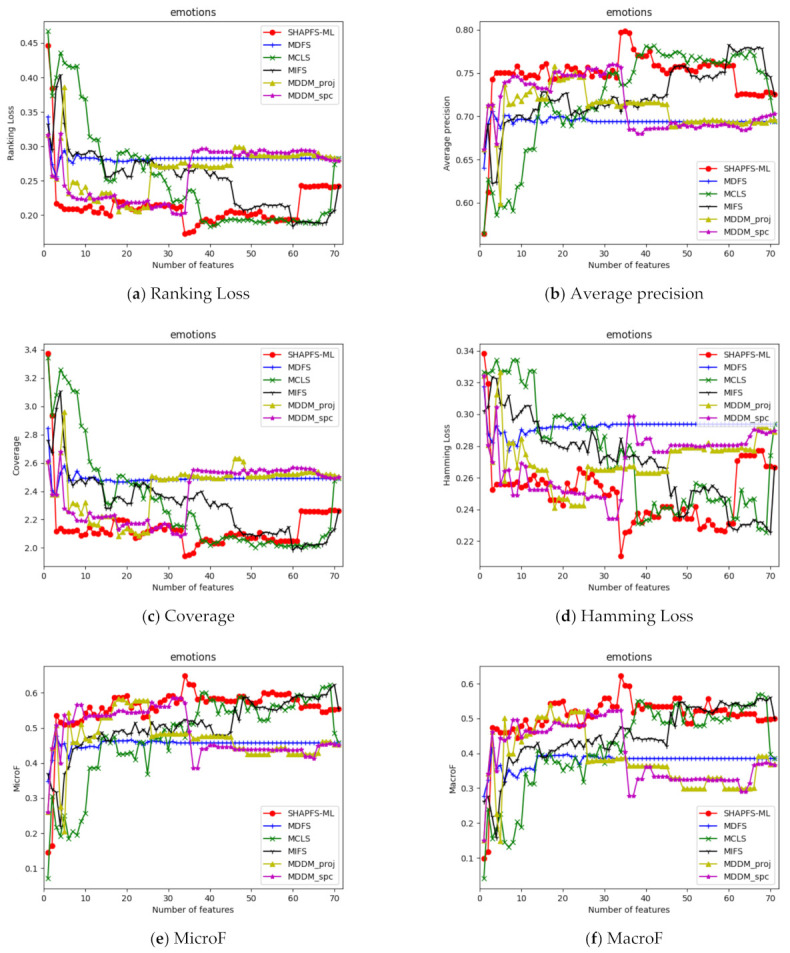
Comparison results with the increase of the number of features under the six indicators on emotions dataset.

**Figure 5:**
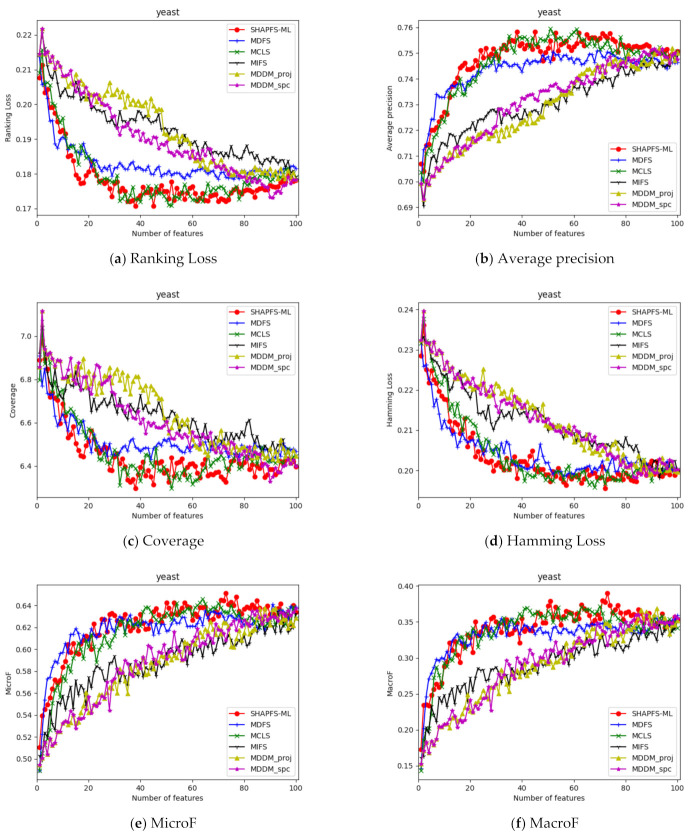
Comparison results with the increase of the number of features under the six indicators on yeast dataset.

**Figure 6:**
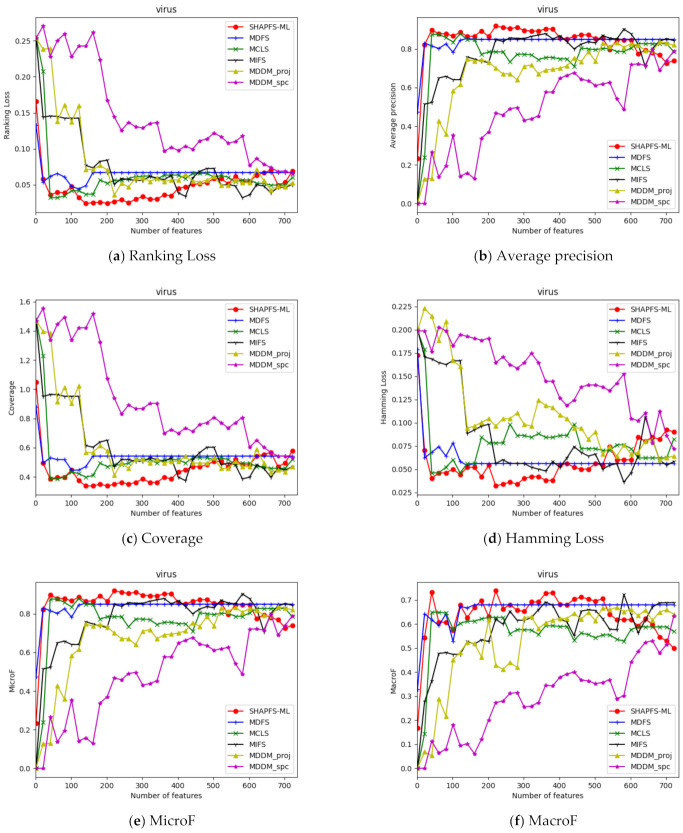
Comparison results with the increase of the number of features under the six indicators on virus dataset.

**Figure 7:**
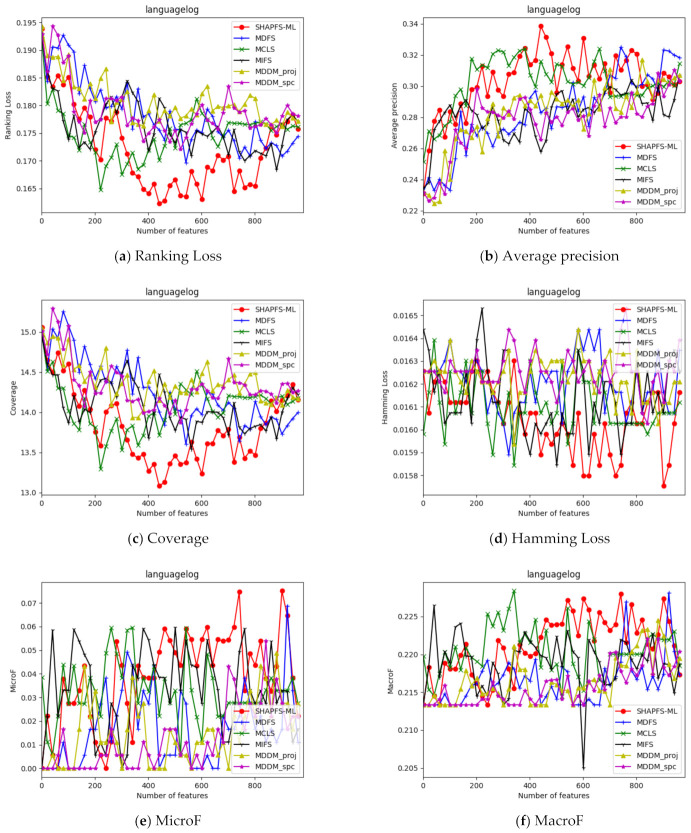
Comparison results with the increase of the number of features under the six indicators on Languagelog dataset.

**Figure 8:**
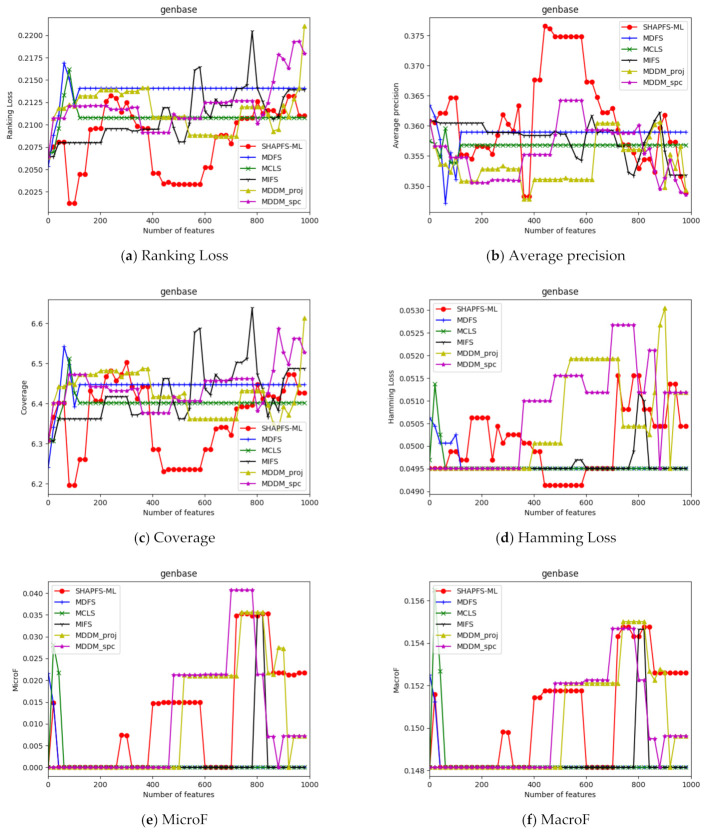
Comparison results with the increase of the number of features under the six indicators on genbase dataset.

**Figure 9:**
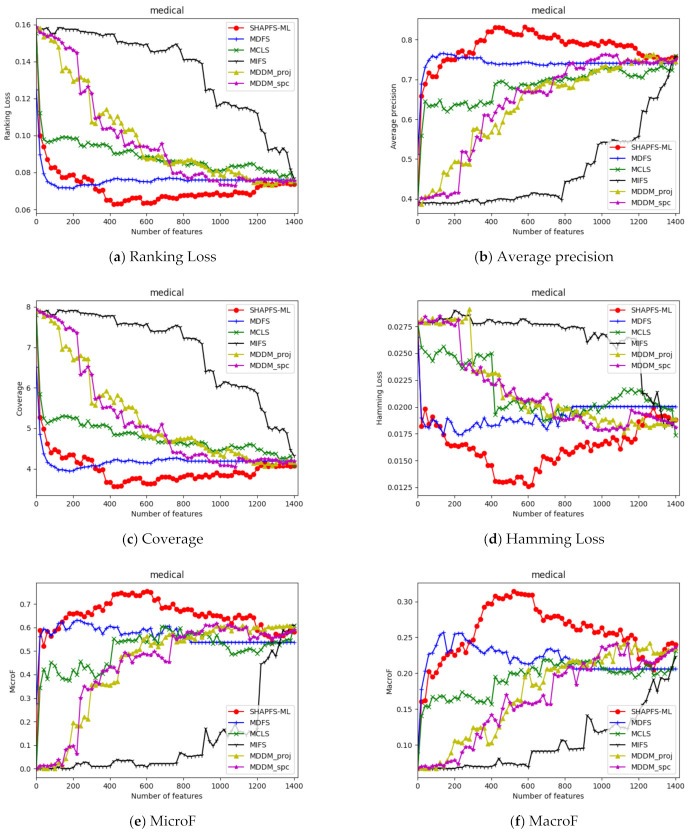
Comparison results with the increase of the number of features under the six indicators on medical dataset.

Figure 3, Figure 4, Figure 5, Figure 6, Figure 7, Figure 8 and Figure 9 show the values of the six algorithms on the seven datasets as the number of features increases on different indicators. From the observation, we can see that SHAPFS-ML has a significant improvement on six indicators compared with other methods on emotions, virus and medical. As the number of features increases, SHAPFS-ML tends to stabilize after reaching a certain maximum value. On yeast and Languagelog, the performance of MCLS is closest to SHAPFS-ML, but SHAPFS-ML can basically obtain the optimal value. On the flags dataset, except for Coverage and MacroF, SHAPFS-ML is slightly inferior to MCLS. On the genbase data set, SHAPFS-ML can obtain the optimal value in addition to the two indicators of MicroF and MacroF.

Through the above analysis, it can be seen that the Shapley value of SHAPFS-ML can effectively evaluate features and identify effective features. In general, non-optimized multi-label feature selection algorithms are more difficult to determine the optimal value, especially for multi-label learning. Under different indicators, the number of features that can obtain the optimal value is different. For a dataset with d dimensions, it is essential to run the classification algorithm d times to obtain the value corresponding to the number of different features on an index. In contrast, the optimization algorithm has excellent global search capabilities and can obtain approximately optimal solutions in one run. According to the experimental results, the features selected by SHAPFS-ML perform better under different multi-label indicators and have better stability.

### 5.6. Complexity Analysis

In this section, the computational complexity of the proposed algorithm is analyzed. When the population size is set to $N_{p}$ and the number of objectives is M, population initialization, crossover operator, mutation operator, and individual fitness calculations all require $O\left(N_{p}\right)$ basic operations. The non-dominated sort requires $O\left(N_{p}log^{M-2}N_{p}\right)$ basic operations. The selection operation of non-dominated individuals requires $O\left(MN_{p}{}^{2}\right)$, so the final computational complexity of the proposed algorithm is max $\left\{O\left(N_{p}log^{M-2}N_{p}\right),\;O\left(MN_{p}{}^{2}\right)\right\}$.

### 5.7. Comparison of Running Time

The running time of the wrapper feature selection algorithm based on the evolutionary optimization depends on the evolutionary algorithm, the size of the data set and the classification algorithm, so it is difficult to measure the actual running time of the evolutionary algorithm [41]. Therefore, we compare the running time of SHAPFS-ML and NSGA III in this section. The running time is the average time of 20 independent runs of each algorithm. It can be seen from Table 10 that the running time of the two algorithms is relatively close, especially on the flags, emotions and virus data sets. SHAPFS-ML is improved on the basis of NSGA III. Both the improved and traditional crossover and mutation operators require linear time.

## 6. Conclusions

Multi-label classification problems are common in real life. In recent years, there have been more studies in the field of multi-label feature selection, but there are few methods based on multi-objective optimization. A wrapper multi-objective optimization feature selection algorithm for multi-label learning fused with Shapley value (SHAPFS-ML) is proposed in this work. This method has two notable properties. First, the idea of Shapley value in game theory is combined with feature selection. We regard feature selection as the process of a cooperative game between features, and an excellent combination of features is selected by evaluating both features and individuals. Secondly, the mutation operator and crossover operator based on Shapley value are proposed to balance the algorithm’s exploration capability and exploitation capability. The experimental results compared with other well-established multi-label feature selection methods on multi-label datasets prove the validity of SHAPFS-ML.

In future work, we will use the Shapley value method to realize feature visualization, and further distinguish relevant features, redundant features and irrelevant features. This research will improve the interpretability of multi-label feature selection algorithms. And we will apply the multi-label feature selection algorithm to a specific problem, such as image annotation.

## Figures and Tables

**Table 1:** Description of datasets.

Dataset	Features	Domain	Labels	Samples	Training	Testing
flags	19	images	7	194	129	65
emotions	72	music	6	593	300	293
yeast	103	biology	14	2417	1500	917
virus	749	biology	6	207	124	83
languagelog	1004	biology	75	1459	1167	292
genbase	1185	biology	27	662	463	199
medical	1449	text	45	978	333	645

**Table 2:** Comparison results of multi-label feature selection in terms of Ranking Loss ↓.

Methods	Flags	Emotions	Yeast	Virus	Languagelog	Genbase	Medical	Avg. Rank
SHAPFS-ML	**0.1915**	**0.1686**	**0.1705**	**0.0146**	0.1629	**0.2028**	**0.0627**	**1.1429**
NSGA III	0.2056	0.1839	0.1751	0.0157	0.1720	0.2044	0.0677	2.8571
MDFS	0.2372	0.2778	0.1794	0.0443	0.1692	0.2131	0.0749	5.1429
MCLS	0.1982	0.2186	0.1765	0.0297	**0.1627**	0.2100	0.0862	3.5714
MIFS	0.2015	0.2546	0.1913	0.0306	0.1706	0.2064	0.1449	4.8571
MDDM_proj	0.2056	0.2050	0.1902	0.0668	0.1779	0.2088	0.0844	5.1429
MDDM_spc	0.2056	0.2013	0.1854	0.1250	0.1810	0.2112	0.0819	5.2857

**Table 3:** Comparison results of multi-label feature selection in terms of Average precision ↑.

Methods	Flags	Emotions	Yeast	Virus	Languagelog	Genbase	Medical	Avg. Rank
SHAPFS-ML	**0.8454**	**0.8002**	**0.7566**	**0.9749**	**0.3158**	**0.3774**	**0.8365**	**1.0000**
NSGA III	0.8225	0.7848	0.7504	0.9703	0.3109	0.3749	0.8138	2.4286
MDFS	0.7929	0.7002	0.7484	0.9291	0.2920	0.3572	0.7412	5.1429
MCLS	0.8284	0.7505	0.7535	0.9478	0.3120	0.3570	0.7022	3.4286
MIFS	0.8154	0.7212	0.7322	0.9438	0.3056	0.3608	0.4149	5.1429
MDDM_proj	0.8182	0.7579	0.7307	0.9000	0.2896	0.3511	0.6940	5.7143
MDDM_spc	0.8182	0.7594	0.7381	0.8275	0.2808	0.3585	0.6988	5.0000

**Table 4:** Comparison results of multi-label feature selection in terms of Coverage ↓.

Methods	Flags	Emotions	Yeast	Virus	Languagelog	Genbase	Medical	Avg. Rank
SHAPFS-ML	**3.6308**	**1.8960**	**6.3108**	**0.2892**	**13.1370**	**6.2111**	**3.5488**	**1.0714**
NSGA III	3.7385	2.0248	6.4046	0.2892	13.7295	6.2412	3.7364	2.9286
MDFS	3.8462	2.4653	6.4526	0.4337	13.5445	6.4271	4.1380	5.0000
MCLS	3.7231	2.1485	6.4820	0.3614	13.1849	6.3920	4.6698	4.2143
MIFS	3.6923	2.3020	6.6249	0.3614	13.6130	6.3065	7.3752	4.6429
MDDM_proj	3.7231	2.0842	6.5540	0.5542	14.0274	6.3618	4.6465	4.9286
MDDM_spc	3.7231	2.0842	6.5267	0.8554	14.5582	6.4171	4.5085	5.2143

**Table 5:** Comparison results of multi-label feature selection in terms of Hamming Loss ↓.

Methods	Flags	Emotions	Yeast	Virus	Languagelog	Genbase	Medical	Avg. Rank
SHAPFS-ML	**0.2352**	**0.2129**	**0.1993**	**0.0301**	**0.0157**	**0.0491**	**0.0137**	**1.0000**
NSGA III	0.2681	0.2170	0.1998	0.0321	0.0158	0.0493	0.0139	2.1429
MDFS	0.3011	0.2921	0.2050	0.3207	0.0162	0.0497	0.0184	4.9286
MCLS	0.2440	0.2639	0.2013	0.2979	0.0163	0.0503	0.0189	4.2857
MIFS	0.2703	0.2698	0.2113	0.3070	0.0159	0.0495	0.0278	5.0000
MDDM_proj	0.3033	0.2409	0.2107	0.3298	0.0162	0.0519	0.0194	6.0000
MDDM_spc	0.3033	0.2343	0.2098	0.3055	0.0160	0.0516	0.0186	4.6429

**Table 6:** Comparison results of multi-label feature selection in terms of MicroF ↑.

Methods	Flags	Emotions	Yeast	Virus	Languagelog	Genbase	Medical	Avg. Rank
SHAPFS-ML	**0.7563**	**0.6446**	**0.6335**	**0.9223**	**0.0853**	0.0149	**0.7246**	**1.4286**
NSGA III	0.7150	0.6362	0.6240	0.9149	0.0649	0.0075	0.7183	2.8571
MDFS	0.6761	0.4636	0.6187	0.7978	0.0201	0.0148	0.5968	5.2857
MCLS	0.7388	0.5114	0.6268	0.8830	0.0165	**0.0357**	0.5929	3.5714
MIFS	0.7036	0.5156	0.6001	0.8601	0.0492	0.0000	0.0218	5.2857
MDDM_proj	0.7000	0.5829	0.5943	0.6848	0.0484	0.0211	0.5751	5.0000
MDDM_spc	0.7000	0.5860	0.6160	0.5122	0.0539	0.0212	0.5627	4.4286

**Table 7:** Comparison results of multi-label feature selection in terms of MacroF ↑.

Methods	Flags	Emotions	Yeast	Virus	Languagelog	Genbase	Medical	Avg. Rank
SHAPFS-ML	**0.6546**	**0.6193**	**0.3649**	**0.7589**	**0.2308**	0.1518	**0.2932**	**1.4286**
NSGA III	0.5279	0.5674	0.3482	0.7062	0.2237	0.1500	0.2778	3.2857
MDFS	0.4777	0.3946	0.3367	0.5084	0.2166	0.1515	0.2144	5.4286
MCLS	0.5657	0.4300	0.3641	0.6115	0.2165	**0.1551**	0.2183	3.5714
MIFS	0.5528	0.4544	0.3033	0.6870	0.2217	0.1481	0.0911	4.8571
MDDM_proj	0.5402	0.4965	0.2950	0.4782	0.2213	0.1521	0.2076	4.5714
MDDM_spc	0.5402	0.5228	0.3227	0.3002	0.2213	0.1521	0.1835	4.4286

**Table 8:** The proportion of selected features.

Methods	Flags	Emotions	Yeast	Virus	Languagelog	Genbase	Medical
SHAPFS-ML	0.2632	0.4583	0.2816	0.3605	0.4781	0.4051	0.4465
NSGA III	0.2105	0.3889	**0.2427**	0.3712	0.4094	0.4203	**0.3823**
MDFS	0.2632	0.2639	0.2816	**0.1669**	0.4452	0.0793	0.4272
MCLS	0.3684	0.4306	0.4466	0.1696	**0.2241**	**0.0194**	0.4976
MIFS	0.6316	0.4583	0.4951	0.2377	0.3924	0.0270	0.4341
MDDM_proj	**0.1053**	**0.2500**	0.4951	0.4099	0.3466	0.4473	0.5003
MDDM_spc	**0.1053**	0.4583	0.5146	0.4686	0.2510	0.3983	0.5003

**Table 9:** HV values of multi-objective algorithms.

**Methods**	**Flags**			**Emotions**		
	**Average**	**Best**	**Worst**	**Average**	**Best**	**Worst**
SHAPFS-ML	**0.6301**	0.7693	0.5375	**0.7326**	0.7781	0.7176
NSGA III	0.5835	0.6237	0.4800	0.5928	0.7453	0.5668
**Methods**	**Yeast**			**Virus**		
	**Average**	**Best**	**Worst**	**Average**	**Best**	**Worst**
SHAPFS-ML	**0.7264**	0.7758	0.6749	**0.5667**	0.6170	0.4612
NSGA III	0.6929	0.7246	0.6184	0.5124	0.5488	0.4392
**Methods**	**Languagelog**			**Genbase**		
	**Average**	**Best**	**Worst**	**Average**	**Best**	**Worst**
SHAPFS-ML	**0.4286**	0.4515	0.4192	**0.3847**	0.3864	0.3807
NSGA III	0.3971	0.4206	0.3734	0.3755	0.3834	0.3506
**Methods**	**Medical**					
	**Average**	**Best**	**Worst**			
SHAPFS-ML	**0.4217**	0.4702	0.3984			
NSGA III	0.3953	0.4410	0.3705			

**Table 10:** The running time of SHAPFS-ML and NSGA III. (S).

**Methods**	**Flags**	**Emotions**	**Yeast**	**Virus**
SHAPFS-ML	85.8350	422.3640	7168.8290	129.0880
NSGA III	89.6130	422.0730	7375.0450	117.2760
**Methods**	**Languagelog**	**Genbase**	**Medical**	
SHAPFS-ML	5569.5000	1112.0010	1840.4340	
NSGA III	5728.6390	1072.4730	1759.2850	

## Data Availability

Not applicable.
